# Predicting intraoperative meningioma consistency using features from standard MRI sequences: a preoperative evaluation

**DOI:** 10.1007/s00701-025-06582-9

**Published:** 2025-06-21

**Authors:** Donata Biernat, Robin Antony Birkeland Bugge, Jon Ramm-Pettersen, Till Schellhorn, Pål Andre Rønning, Eirik Helseth, Kyrre Eeg Emblem, Karoline Skogen

**Affiliations:** 1 Department of Radiology, Division of Radiology and Nuclear Medicine, Oslo University Hospital, Oslo, Norway; 2 Department of Neurosurgery, Oslo University Hospital, Oslo, Norway; 3 Department of Physics and Computational Radiology, Division of Radiology and Nuclear Medicine, Oslo University Hospital, Oslo, Norway; 4 Institute of Clinical Medicine, Faculty of Medicine, University of Oslo, Oslo, Norway

**Keywords:** Meningioma, Tumor, Consistency, MRI

## Abstract

**Background:**

Symptomatic meningiomas may require surgical resection to save or improve neurological function. The extent of tumor resection depends on multiple factors, including the tumor's consistency, its location, and the patient's overall condition. This prospective study aims to explore new criteria in combination with previously proposed tumor features on MRI to establish a rapid approach to tumor consistency characterization pre-operatively.

**Methods:**

Forty-eight patients with meningiomas were prospectively included and underwent a dedicated MRI protocol prior to surgery. Qualitative and quantitative MRI characteristics of the tumor were correlated to a previously proposed surgical tumor consistency grading.

**Results:**

Soft tumors were associated with homogeneous contrast enhancement, high T2 signal, absence of peritumoral edema (PTE), the presence of tumor cysts, and a uniformly dark appearance on fractional anisotropy (FA) maps. In contrast, firmer tumors were characterized by heterogeneous contrast enhancement, low T2 signal, the presence of PTE, absence of tumor cysts and a heterogeneous appearance on FA maps, requiring supranormal ultrasonic aspirator settings. Tumor signal quantification on T2 and Apparent Diffusion Coefficient maps (ADC) correlated moderately to tumor consistency. T1 sequences did not contribute in determining tumor consistency.

**Conclusion:**

An array of simple qualitative meningioma characteristics on MRI can assist in swift discrimination of soft and hard tumors preoperatively. These have been displayed in a figure that can easily be implemented clinically for optimal surgical planning.

**Supplementary information:**

The online version contains supplementary material available at 10.1007/s00701-025-06582-9.

## Introduction

Meningiomas are the most common primary intracranial tumors in the adult population. When surgery is indicated, primarily due to tumor growth or symptoms caused by adjacent brain and nerve compression, the aim of surgery is usually complete resection of the tumor, including its attachments [20]. Magnetic resonance imaging (MRI) is mandatory to provide the exact anatomical position of the tumor and proximity to surrounding structures, facilitating optimal surgical planning during surgery [6].

Tumor location and tumor consistency are critical factors influencing the risks of surgery, and thus the prospect of complete tumor resection. Soft tumors are often readily debulked with low suction intensity by an ultrasonic aspirator before dissecting tumor attachments, ultimately resulting in shorter operative time. Harder tumors pose an increased risk of operative morbidity, as they often require greater suction intensity during resection. This elevated force can increase the likelihood of damage to surrounding structures and prolong the duration of the surgery. Therefore, accurately predicting tumor consistency preoperatively could significantly aid in surgical planning and patient counselling, helping to mitigate these risks and improve patient outcomes [18].

Historically, using MRI to predict meningioma characteristics, including consistency, has demonstrated diverging results. T1 and T2 weighted images, hereafter referred to as T1 and T2, are the most studied [3, 5, 11, 14, 16, 23–25], but advanced MR sequences such as diffusion weighted imaging (DWI) [10] diffusion tensor imaging (DTI) [2, 7, 13] and perfusion imaging (PWI) have also been explored. Previous studies used different qualitative and quantitative MRI grading systems to predict surgical tumor consistency, again with diverging results. Intraoperative assessment of surgical tumor consistency is referred to as the gold standard. However, a universally accepted grading scheme does not exist, as tumor resection techniques vary between institutions, making comparisons difficult. In order to communicate meningioma consistency, Zada et al. proposed a 5 point intraoperative system to grade meningioma consistency based on the tissue’s resistance to suction [27].

This prospective study aims to establish a simple and rapid method for visually evaluating soft vs hard meningioma consistency on preoperative MRI sequences, by combining simple tumor characteristics visible on T1, T2, DWI, ADC, and DTI. Tumor characteristics are analyzed and correlated to the systematic intraoperative tumor consistency evaluation scheme proposed by Zada [27].

## Materials and methods

### Study set up and general characteristics

Sixty-seven symptomatic patients with suspected meningioma were prospectively included between September 2020 and December 2022. Inclusion criteria were adult patients (> 18 years old) with MRI findings suggestive of an intracranial meningioma ≥ 3 cm diameter and planned craniotomy. All tumors were postoperatively verified as meningiomas (WHO grade I-III). The study was approved by the Regional Research Ethics Committee (REC) and the Institutional Review Board (REK 20446 (2017/1875)), and patient`s written consent was collected. Nineteen patients were excluded due to lack of surgical evaluation (9) or incomplete MRI (10) resulting in a total of 48 included patients. The following general patient and tumor data were collected: sex, age, tumor and peritumoral edema (PTE) volume in cm^3^, tumor location, grade and histology. Other tumor characteristics included qualitative evaluation of tumor signal on T1, T2, DWI and FA, tumor heterogeneity, tumor and capsule enhancement patterns on T1 with contrast (T1c), regularity of tumor capsule, and invasion of bone and dural venous sinus. Described in detail below are the systematic qualitative and quantitative radiological assessments on anatomic and advanced MRI sequences.

### Image acquisition and processing

All patients underwent a dedicated preoperative MRI protocol on a 3 T MR scanner (Signa Premier, GE Medical Systems, Milwaukee, Wisconsin) (Table 1), using a 32-channel head coil. The MRI sequences included T1, T2, 3D T2, T1c and DTI deriving DWI, ADC and FA maps. Tumor volume and PTE were semi-automatically segmented on post-contrast T1c and 3D T2 respectively, using BrainLab elements software (Softbrush 4.0) by an experienced neuroradiologist (DB).

**Table 1:** MRI tumor protocol

MRI protocol											
Sequences	MRI parameters										
	Name	TR	TE	TI	FOV	FLA	ST	SS	Dir	ARC Phase/Slice	HS
T1	Sag MPR	700	2,5	900	25.6	8	1.0	0	NA	1.80/1.20	NA
T2	Ax TSE	9447	117.2	NA	24.0	142	3.0	0.3	NA	2	NA
T2	Sag 3D	3000	75	NA	25.6	NA	1.0	0	NA	2/2	1.2
T1c	Sag MPR	700	2.5	900	25.6	8	1.0		NA	1.80/1.20	NA
DTI	Ax	3000	min	NA	24.0	NA	4.0	1.0	27	3	NA

*ARC*; Autocalibrating Reconstruction for Cartesian Imaging, *Dir*; Directions, *DTI*; Diffusion Tensor Image, *FLA*; Flip Angle, *FOV*; Field of View, *HS*; Hypersence, *MPR*; MP Rage, *SS*; Slice Space, *ST*; Slice Thickness, *TE*; Time to Echo (ms), *TI*; Inversion Time (ms), *TR*; Time to Repetition (ms), *T1*; T1 weighted images, *T2*; T2 weighted images, *T1c*; T1 weighted images with contrast

### Radiological tumor assessment

The radiological tumor consistency assessment was performed qualitatively and quantitatively.

Two neuroradiologists, with 10 (DB) and 16 (TS) years of experience respectively, evaluated the MRI properties of the meningioma. CT caput was not consistently performed preoperatively and not used for the assessment.

### Qualitative tumor assessment

**Fig. 1:**
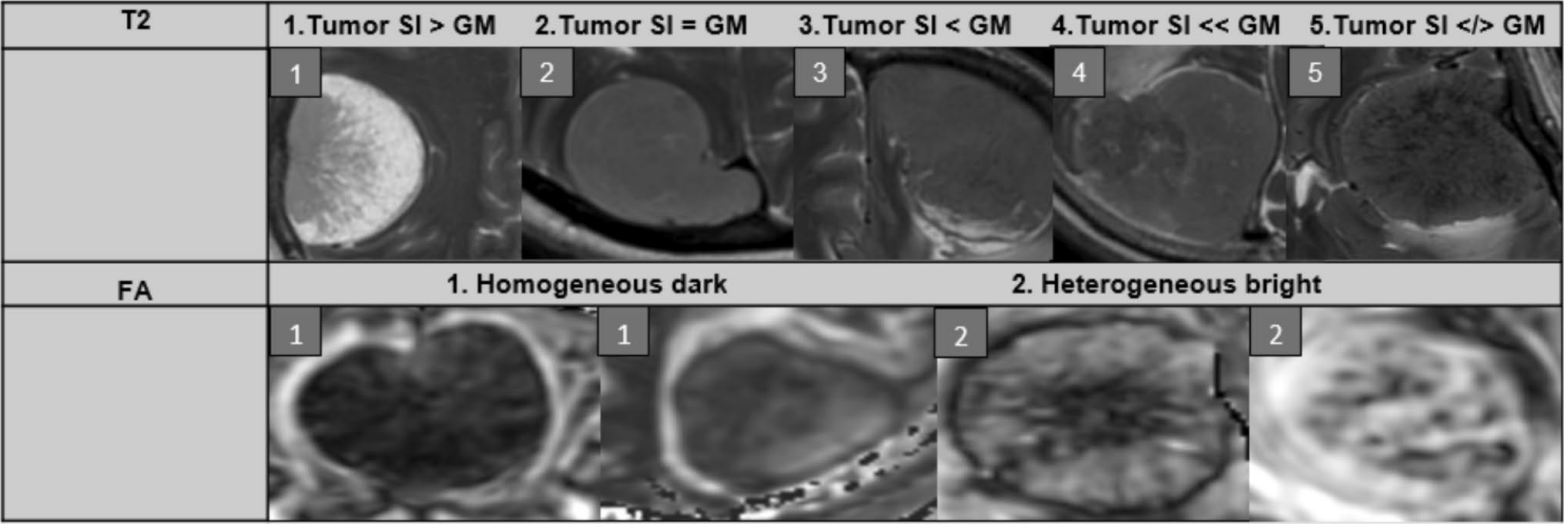
Radiological tumor signal grading on T2 and FA as explained above. T2 column shows the visual range of tumor signal, (1- higher signal than contralateral insular grey matter (cGM) to 5- signal considerably lower than cGM). FA map column shows variation in FA appearance (1-uniform dark, vs 2-heterogenous, mosaic like, bright)

The primary tumor assessment was done by neuroradiologist 1 (DB) preoperatively, at the time of the MRI study, while the second assessment was performed by neuroradiologist 1 and 2 after study inclusion in the following manner: Apparent tumor signal in four tumor quadrants (anterior, posterior, superior and inferior), was visually assessed by comparing tumor signal to contralateral insular grey matter signal (cGM) on T1, T2 and 3D T2. Tumor signal grading was adapted according to the previously published neurosurgical grading system by Zada et al.[27] and varied from 1 to 5. Tumor grade 1 corresponded to lower tumor signal on T1 and higher signal on T2 than cGM, exemplified in Fig. 1, and was assumed to represent very soft tumor consistency. Grade 2 corresponded to similar tumor signal as cGM and was assumed to represent soft tumor consistency. Tumor signal slightly and considerably higher on T1 and lower on T2 was assigned grade 3 and 4 respectively, assumed to represent a spectrum of intermediate and hard tumor consistencies, respectively. Grade 5 was assigned to heavily heterogeneous tumor, where simple scaling did not apply, and was assumed to be hardest tumor consistency. In addition gross DWI signal intensity of tumor relative to cortical grey matter was defined as 1- hyperintense, 2- isointense, 3 – hypointense and 4 – heterogeneous tumors with focal signal hypointensity. Tumor appearance on FA maps was visually assessed as 1- homogenous dark or 2-heterogeneous mosaic like appearing tumor (Fig. 1).

### Quantitative tumor assessment

Quantification of T1 and T2 signal was performed by manually drawing largest in-plane ROI centrally in the tumor. Other 1 cm ROIs, were drawn in the contralateral white matter (cWM), contralateral cerebellar peduncle (cCP) and a 3 mm ROI in contralateral insular cortical grey matter (cGM). Normalization of tumor values on T1 and T2 was performed by dividing mean tumor ROI by mean cWM ROI. Using these different ROIs, the following previously published parameters on T1 and T2 were calculated; mean tumor ROI, tumor/cCP ratio, tumor/cGM ratio and subtracted cGM/tumor ratio (cGM ROI – tumor ROI) [1, 17, 25]. ADC ROI was established by calculating the mean of ROI in four tumor quadrants.

### Systematic neurosurgical data collection and evaluation

Neurosurgeons were not blinded to the primary preoperative standard radiological report, but they were blinded to the radiologist`s qualitative and quantitative tumor assessment. Seven operating surgeons, all with an experience of minimum 8 years (8–20 years), assessed intraoperative tumor consistency according to the previously reported surgical grading system [27]. In concordance with the radiological grading, tumor was divided into an anterior, posterior, superior and inferior part, and tumor consistency was evaluated according to resistance to aspiration on Calverton Ultrasonic Surgical Aspirator (CUSA® Clarity, Integra LifeSciences). The grading range was similar to the radiological grading system, dividing tumor consistency into 1–5, where 1- was equal to soft tumor removable at lowest resistance to aspiration, 2—represented usage of CUSA at low power (CUSA 0–40), 3—intermediate tumor consistency represented CUSA with power 40–60, 4—CUSA power at 60–90 and need of cautery loop, and 5—represented hard tumor consistency with CUSA at 100 and/or the need of scissors, knife, or other surgical resection equipment. The surgeons were instructed to set CUSA at the lowest possible setting necessary to debulk the tumor.

Finally, surgical tumor consistency was established by calculating mean tumor consistency (mean of four quadrants) with further subcategorization into soft (surgical consistency 1, 2), and firm tumors (surgical consistency 3, 4, 5), requiring CUSA a power setting below or above 40. In the postoperative setting WHO tumor grade, Ki-67 count, and Simpson resection grade was collected.

### Statistical analysis

Statistical analysis was performed using the Stata 18 software package (StataCorp. 2023. *Stata Statistical Software: Release 18*. College Station, TX: StataCorp LLC). Cohen’s Kappa was used to assess intra- and inter-rater agreement between the two radiologists. Calculation of Spearman’s correlation coefficient (r) was used to establish the association between soft (1,2) and firm (3,4,5) tumor consistency and various qualitative and quantitative tumor characteristics. ROC analysis was performed for quantitative T1, T2 and ADC analysis.

The strength of association between different qualitative tumor characteristics and actual tumor consistency was established by calculating Odds ratios (OR). Uni- and multivariable logistic regression were used to investigate the effect of different covariates on tumor consistency. All significance levels were set at *p* ≤ 0.05.

## Results

### Clinical characteristics

Over the course of 27 months, 48 patients were included. The mean age of the study population was 63 years and consisted of 40 women (83%). Mean tumor volume was 41 ml (range 7–96 ml). Thirty-eight tumors were WHO grade 1 meningiomas (80%), 9 were atypical WHO grade 2 (18%) and 1 was a WHO grade 3 (2%) tumor.

Thirty-seven patients presented with peritumoral edema (PTE) (77%). The neurosurgical ground truth assessment of tumor consistency showed one very soft—grade 1 tumor (2%), 12 moderately soft tumors—grade 2 (23%), 17 intermediately firm tumors—grade 3 (37.5%), 17 firm—grade 4 (35.5%) and one hard—grade 5 tumor (2%). Further patient demographics and tumor characteristics are presented in Table 2.

**Table 2:** Radiological tumor properties grouped according to surgical tumor consistency

	Meningioma consistency		
Parameters			
	Soft	Hard	Total (%)
	1,2	(3,4,5)	
	*n* = *13 (35%)*	*n* = *35 (65%)*	*n* = 48 (100%)
**Tumor volume, cm3 (mean**)	42	40	40
Location			
Convexity	6	8	14 (29)
Falx	1	9	10 (20)
Parasagittal	2	5	7 (15)
Clinoid	2	4	6 (13)
Sphenoidal	1	5	6 (13)
Olphactorius	1	3	4 (8)
Other	0	1	1 (2)
**T1 WI signal, n (%)**			
Hypointense	3	10	13 (27)
Isointense	9	24	33 (69)
Hyperintense	1	1	2 (4)
**T2 WI signal, n (%)**			
Hypointense	1	15	16 (33)
Isointense	5	14	19 (40)
Hyperintense	7	6	13 (27)
**DWI signal, n (%)**			
Hypointense	1	6	7 (15)
Isointense	4	17	21 (44)
Hyperintense	8	12	20 (41)
**Heterogeneity**			
Yes	8	27	35 (73)
No	5	8	13 (27)
Cyst	7	6	13 (27)
Vasculature	4	23	27 (56)
Calcification	6	11	17 (35)
Enostotic spur	7	14	21 (44)
**Tumor contrast enhancement**			
Homogeneous	9	6	15 (31)
Heterogeneous	4	29	33 (69)
**Tumor capsule enhancement**			
Yes	11	22	33 (69)
No	2	13	15 (31)
**Tumor surface**			
Smooth	5	7	12 (25)
Microlobular	5	20	25 (52)
Macrolobular	3	8	11 (23)
**Edema**			
Yes	6	29	35 (73)
No	7	6	13 (27)
**Invasion**			
Bone	2	11	13 (27)
Dural sinus	2	11	13 (27)
Cortical veins	9	30	39 (81)
**FA**			
Homogeneous dark	5	4	9 (19)
Heterogeneous bright	8	31	39 (81)

### Tumor consistency assessment

**Fig. 2:**
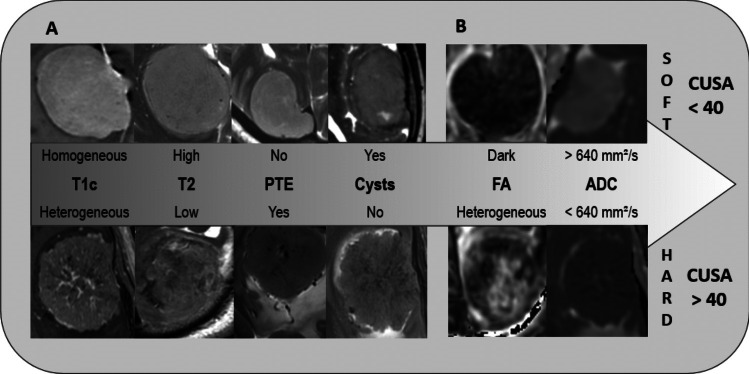
Qualitative meningioma characteristics predictive of soft (1, 2,) and hard (3, 4, 5) tumor consistency on univariate regression analysis. Homogenous contrast enhancement, high T2 signal, absence of PTE, presence of tumor cysts, combined dark uniform FA, and ADC ROI > 6.4 × 10^–4^ mm2/s were strongly predictive of soft tumors, while heterogeneous contrast enhancement, low T2 signal, presence of PTE, absence of tumor cysts, heterogeneous FA along with ADC ROI < 6.4 × 10^–4^ mm2/s were predictive of intermediate and hard tumors, requiring CUSA suction power of 40 or more to remove tumor. The strength of association shown in Table 4

**Fig. 3:**
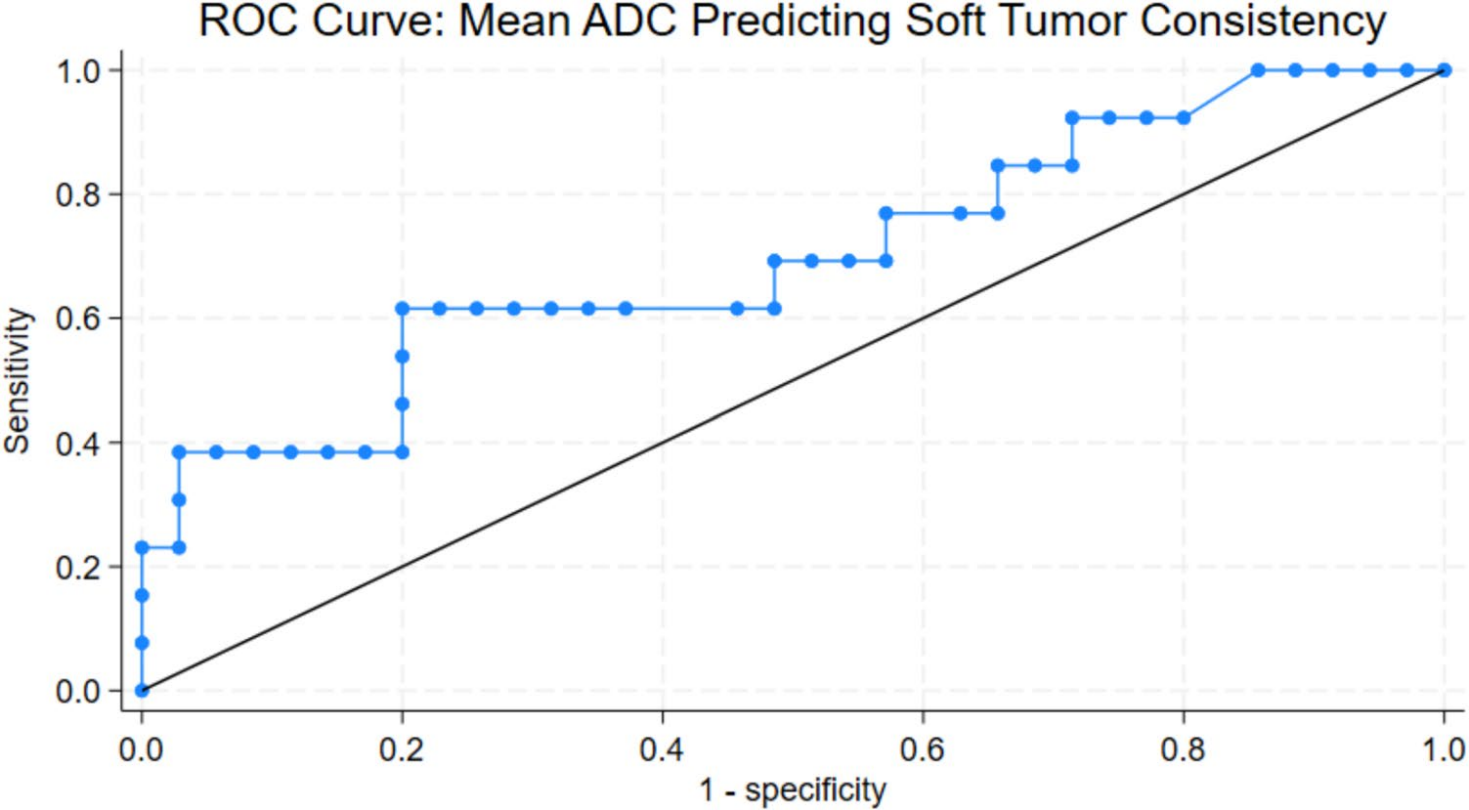
Receiver operating characteristic (ROC) curve showing the diagnostic performance of ADC values from mean tumor ROI in predicting tumor consistency. The area under the curve (AUC) was 0.71, indicating moderate diagnostic accuracy at threshold 0.64 × 10^–4^ mm^2^/s, with sensitivity 62%, specificity 80%. Higher values being indicative of soft tumors

When we stratified the intraoperative ground truth tumor consistency into soft (1, 2), and hard (3, 4, 5) the following qualitative tumor characteristics were significantly correlated to soft surgical tumor consistency in univariate regression analysis, with elevated OR (Table 3, Table 4); homogeneous appearance on T1c, T2 hyperintensity, the absence of peritumoral edema (PTE), presence of small intratumoral cysts and homogenous dark appearance on FA maps. Conversely, hard tumors were associated with heterogeneous appearance on T1c, T2 hypointensity, presence of PTE, absence of intratumoral cysts and heterogeneous appearance on FA maps (Fig. 2). The ROC analysis of mean ADC ROI showed a threshold > 6.4 × 10^–4^ mm2/s to predict softer tumor consistency and vice versa for the hard tumors with a sensitivity and specificity of 62% and 80%, respectively (AUC 0.71), (Fig. 3).

**Table 3:** Univariate and multivariate analysis of visual tumor characteristics predicting soft tumor consistency

Predictor of Soft Tumor	Univariate β (95% CI)	p-value	Multivariate β (95% CI)	p-value
Homogeneous enhancement	0.50 (0.22, 0.78)	0.001	0.42 (0.15, 0.69)	0.003
Cysts	0.35 (0.09, 0.61)	0.01	0.31 (0.06, 0.57)	0.016
FA	0.35 (0.03, 0.67)	0.03	0.17 (−0.11, 0.46)	0.23
No edema	0.36 (0.09, 0.64)	0.01	0.18 (−0.09, 0.45)	0.19
T2 Hyperintensity	0.29 (0.20, 0.56)	0.03	−0.02 (−0.29, 0.25)	0.89

**Table 4:** Strength of assosciation, shown by Odds ratio (OR) between radiological tumor parameters and surgical tumor consistency

Parameter	Tumor consistency				
	Soft	Hard	OR	p-value	CI
T1c	Homogeneous	Heterogeneous	10	0.001 *	2.5–47
T2 signal	High	Low	9	0.04 *	1.0–77
PTE	No	Yes	5.6	0.016 *	1.3–22.9
Cysts	Yes	No	4.1	0.047 *	1.02–16
FA	Dark	Heterogeneous	4.8	0.04 *	1.05–22.3

*CI*; Confidence Interval, *FA*; Fractional Anisotropy, *OR*; Odds Ratio, *PTE*; Peritumoral Edema, *T1c*; T1 with contrast, * significant values

In a multivariate regression analysis only homogeneous contrast enhancement (p = 0.003) and the presence of cysts (p = 0.01), were strongly correlated to softer tumors as shown in Table 3, while the absence of tumor cysts (p = 0.007) and heterogeneous contrast enhancement were strongly correlated with hard tumors (p = 0.004). PTE, T2 signal intensity and homogeneity of FA appearance did not reach significance.

We found no correlation between overall tumor consistency and presence of general tumor heterogeneity on T2, DWI signal, tumor capsule, tumor surface, invasion of bone or dural sinus, vascularity, PTE volume, WHO grade, or Simpson resection grade.Tumor grade correlated moderately with Ki-67 count (r = 0.4, p = 0.007), but was not correlated to tumor consistency (r = 0.08, p = 0.5).

### Qualitative tumor assessment agreement

We found overall moderate and high intra-rater agreement of qualitative radiological tumor assessment of quadrant (Kappa = 0.5), mean (Kappa = 0.6) and categorical tumor consistency (Kappa = 0.8). Inter-rater radiological agreement was low for quadrant and mean tumor consistency assessment (Kappa = 0.1), and moderate for categorical (soft vs hard) tumor consistency assessment (Kappa = 0.4). Inter-rater agreement between radiologist and neurosurgeons was consistently low for quadrant (Kappa = 0.1), mean, and for categorical tumor assessment (Kappa = 0.2) for T2 (Table 5), and nonexistent for T1.

**Table 5:** Showing intra-rater agreement for radiologist 1(*) and inter-rater agreement for radiologist 1 and 2 (**) for quadrant (Q), mean and categorical T2 tumor signal assessment, and inter-rater agreement for radiologist 1 and surgical tumor consistency assessment preoperatively (***), when compared on quadrant (Q), mean and categorical level

	Kappa	p-value
Intra-rater agreement T2 MRI SI (*)		
Q vs Q	0.5	0.01–0.2
mean	0.6	0.002
categorical	0.84	0.0003
Inter-rater agreement T2 MRI SI (**)		
Q vs Q	0.08	0.01–0.2
mean	0.16	0.002
categorical	0.45	0.0003
Inter-rater agreement T2 MRI SI vs surgery (***)		
Q vs Q	0.1	0.01—0.2
mean	0.2	0.08
categorical	0.2	0.01

### Quantitative tumor assessment agreement

Quantitative analysis of normalized mean tumor ROI on T2 showed moderate correlation to surgical consistency for softest and hardest tumors (r = 0.3, p = 0.03, r = −0.3, p = 0.03, respectively). ROC analysis found a sensitivity of 69% and specificity of 69% at 1.72 ratio with an AUC of 0.69, higher values being indicative of soft tumors and lower values indicative of firm tumors. We found a moderate correlation between surgical tumor consistency and quantitative T2 tumor/cCP ratio (r = 0.31, p = 0.02) with sensitivity 62%, specificity 80% at cutoff 1.74 with AUC of 71, higher values being indicative of soft tumor (Supplementary material, Fig. 4, Fig. 5). On ADC map we found moderate correlation between mean tumor ROI and soft tumor consistency (r = 0.3, p = 0.03) with sensitivity 62%, specificity 80% at a threshold of 0.64 × 10^–4^ mm^2^/s, with AUC 0.71, higher values being indicative of soft tumors (Fig. 3).

## Discussion

In this study we focused on validating previously proposed MRI characteristics of meningioma consistency and additionally define simple new MRI tumor properties, which can help in predicting meningioma consistency preoperatively. Due to the heterogeneity of these tumors, combining these properties facilitates discrimination of soft and hard meningiomas more accurately than sole tumor assessment on one sequence. This study focused on combining MRI features in a visually clarifying figure with decreasing order of significance from left to right (Fig. 2). The figure depicts heterogeneity of contrast enhancement on T1c as the most significant discriminating feature between soft and hard tumors, in addition to tumor signal intensity on T2, PTE and cysts. All these parameters are easily assessed on the routine MRI sequences T1c and T2. More advanced sequences, such as DWI and DTI, offer less discriminating power compared to T1c or T2.

The structural composition of meningiomas can be physiologically heterogeneous, varying from cystic to fibrous and calcified composition. CT is the modality of choice to visualize macro-calcification of tumor tissue, but unfortunately, it has limited ability to discriminate the nature of other tissue, such as collagenous or fibrous tumor composition, associated with firm tumor consistency. MRI sequences can elicit these features. T1c highlights tissue enhancement. This study finds T1c to be the strongest single discriminator of tumor consistency indicating homogeneous enhancement to represent soft tumors and heterogeneous enhancement to indicate a harder tumor consistency, potentially due to reduced enhancement of fibrous tissue strands accompanied by micro-calcification and absence of enostotic spurs and a vascular core [12]. There is an overlap where some enhancement characteristics can be misleading, as soft meningiomas with cysts will appear heterogeneous after contrast enhancement. This highlights the necessity for combining T1c and T2 sequences for consistency assessment (Fig. 2).

The T2 sequence visualizes nuances in tissue water content, indicating the higher the T2 signal, the higher the presumed fluid concentration, thought to represent soft consistency. Decreased T2 signal is presumed to represent more fibrous tissue, suggesting a harder consistency. Our results align with this hypothesis that high signal indicates soft and low signal hard meningiomas on T2, respectively. These results have also been reported in other studies [25]. To our knowledge only one study found no correlation between T2 signal and consistency [13]. Symptomatic tumors tend to be large and often heterogeneous. Visual tumor heterogeneity apart from water and collagen content variation may represent vasculature, calcification and blood, all of which can appear with similar signal on T2 and are presumably one reason for diverging previous results. Subjectivity of signal assessment may also be of significance (Table 5). Hence, using solely T2 signal to assess tumor consistency can pose challenging.

PTE is often present and correlating meningioma consistency with PTE has been investigated previously, consistently failing to show a significant correlation [13, 16]. In this study 77% of the tumors presented with edema, which was observed in all tumor consistencies (r = 0.3, p = 0.03), but was more common in firm tumors (r = 0.4, p < 0.01), as shown in Table 2. The exact explanation for this is not understood. Kim et al. found irregular tumor margins, tumor surface proteins [8, 16], adherence to underlying brain and absence of arachnoid plane to be consistent with PTE. Tumor size has also been shown to correlate with PTE [14, 15]. This was not supported in our study as the largest tumors in our study had a lower incidence of PTE compared to the harder but smaller tumors, again highlighting the multifaceted causality of edema formation. Recent insight into the glymphatic clearance system has led to the hypothesis of PTE formation due to glymphatic dysfunction in fast growing meningiomas, possibly reflecting high grade meningiomas [21]. Our study does not support this hypothesis. We observe a tendency of soft large tumors of all WHO grades presenting more commonly without PTE, as opposed to the smaller, but harder tumors. We hypothesize that hard tumor pressing on perifocal structures may contribute to accelerated glymphatic dysfunction in the elderly population, but more insight is needed on this topic. Another possible reason for this observation could be discrepancies in defining soft vs hard tumors. We define soft tumors requiring a CUSA power of 40 or more for removal.

Few studies have considered intratumoral cysts as a feature to discriminate between soft and hard tumor consistencies, potentially due to varying definition of cyst appearance on MRI. One study reported a low incidence of large intratumoral cysts [4], leading to a low correlation between cysts and tumor consistency. When defining cysts as any focal, non-enhancing intratumoral fluid filled lesion of varying sizes, presenting hyperintense on T2 and hypointense on T1, this study found an increased incidence of soft (1,2) tumors. None of the hardest tumors (4,5) had cysts.

All of the above-mentioned features can easily be assessed on routine MRI sequences, T2 and T1c.

The FA maps derived from DTI are thought to represent the architectural nature of meningiomas by measuring anisotropic movement, imitating white mater tracts in the brain. One histologic characteristic thought to contribute to consistency of meningiomas are fibrotic strands [19]. Previous studies have implied this could be defined by anisotropic movement along fibrous strands in the tumor, appearing heterogeneous (mosaic-like) on FA maps in hard tumors [2, 7, 13, 19, 22]. Lack of these fibrous strands could support the homogenously dark-appearing FA maps often seen in softer meningiomas, found in this study, while more heterogeneous FA appearance is an indicator for firmer, more fibrotic and heterogeneous tumors (Table 2). Other studies have also shown similar tendencies. [2, 7, 13, 19, 22]

DWI using B1000 and ADC maps are known to reflect tissue cellularity and integrity. High cellular density and disruption of cellular membranes typically appear with increased signal on DWI, and decreased values (low signal) on ADC maps. Increased cellularity has been correlated to firmer and higher grade brain tumors such as gliomas and meningiomas [26], but there is currently limited and contradictory data correlating DWI signal and ADC values to meningioma consistency[23, 26]. Limpastan et al. reported a strong correlation between firm tumors and decreased T2 signal and DWI intensity (B1000), which is somehow counterintuitive if increased cellularity is a predictor of firm tumor consistency [9]. Low signal on T2 and DWI may thus reflect the existence of fibrous or calcified tumor composition, regardless of tumor cellularity. Our study supports the finding of increased T2 in soft tumors, but did not reach significance for DWI signal in the same tumors. ADC values and their quantification shows opposing results in the literature, where Phuttharak et al. [12] found a strong correlation of high ADC values and Yogi et al. [26] found reduced ADC values in hard tumors. Quantification bias can be a reason for this discrepancy, as Phuttarak et al. used only one small size ROI in large heterogeneous tumors. In concordance with Yogi [26] we find moderate correlation between intermediate and hard tumor consistency and ADC values, at the same threshold of 6.4 × 10^–4^ mm2/s or lower.

In a multivariate regression analysis homogeneous contrast enhancement, and presence of cysts were predictive of soft tumors. Heterogeneous contrast enhancement, and the absence of tumor cysts were significantly correlated to hard tumor consistency. Absence of PTE, T2 signal intensity and FA appearance did not reach significance for tumor consistency but show a clear trend on univariate analysis. The lack of significance for PTE and T2 signal in multivariate analysis highlights the heterogeneity within these tumors. We interpret this finding reflective of the importance of multifactorial approach to meningioma assessment. Using multiple criteria, as exemplified in Fig. 3, may add valuable supplementary information in the preoperative tumor consistency assessment.

Tumor consistency assessment by T1 and T2 signal compared to grey matter alone was not reliable due to the high variability for qualitative signal assessment by two experienced radiologists and the lack of correlation with surgical findings (Table 5). Likewise, the quantification of tumor signal from the different anatomic sequences, normalization, and further application of ROIs in various, previously published tumor/brain ratios to discriminate between the two consistencies was cumbersome and presents consistency difficulties. In this series, moderate correlation between normalized T2 tumor/cCP ratio and mean T2 and ADC ROI was observed (Supplementary material, Fig. 4 and 5), but implementation of ratio calculation into clinical practice seems difficult. As previous studies have shown, no individual MRI sequence or quantitative measurement is sufficient to confirm tumor consistency. Combining sequences and tumor characteristics, in a visually pleasing figure, is practical and constitutes a simple and accessible method to distinguish between soft and hard tumor consistency that can easily be implemented into the clinical routine and surgical planning.

## Study limitations

A limitation of our study is the relatively small population size, but it constitutes a representative population with regards to age and sex. The prospective nature of this study and a tumor size ≥ 3 cm could have been a reason for this low number. However, the importance of a larger tumor to display an array of dominating features better has shown to be important for determining tumor consistency. Another limitation is a potential reporting bias in the surgical report, performed by seven different surgeons to conclude on tumor consistency. To reduce potential surgical subjectivity, a structured surgical report was performed. There was only one experienced neuroradiologist drawing the ROIs for both the quantitative, ratio and normalization assessments, which leaves room for bias. In order to reduce this bias we used previously published methods. Finally, even though this is a prospective study, unfortunately a correlation between radiological features and histopathology was not performed, this would have strengthened these findings. Validation of these radiological features is recommended for future studies together with histological correlate.

## Conclusion

Integrating an array of MRI features from routine sequences enhances the ability to predict meningioma consistency preoperatively. This study identifies T1c enhancement patterns, in addition to T2 signal, PTE and intratumoral cysts to be the most significant features. Homogeneity of FA appearance and mean ADC ROI values had a supporting contribution in assessing meningioma consistency.

## Supplementary Information

Below is the link to the electronic supplementary material.

ESM 1 (PDF 171 KB)

## Data Availability

No datasets were generated or analysed during the current study.

## Abbreviations

ADC: Apparent Diffusion Coefficient
cWM: Contralateral white matter
DWI: Diffusion Weighted Imaging, B1000
PTE: Peritumoral edema
ROI: Region of interest
Subtracted cGM/tumor ratio: Contralateral mean insular grey matter ROI / mean tumor ROI ratio
T1: T1 weighted images
T2: T2 weighted images
T1c: T1 weighted images with contrast
Tumor/cGM ratio: Tumor /contralateral insular grey matter ratio
Tumor/cCP ratio: Tumor / contralateral cerebellar peduncle ratio

## References

[1] Alyamany M, Alshardan MM, Jamea AA, ElBakry N, Soualmi L, Orz Y (2018) Meningioma consistency: correlation between magnetic resonance imaging characteristics, operative findings, and histopathological features. Asian journal of neurosurgery 13:324–328. 10.4103/1793-5482.228515 29682029 10.4103/1793-5482.228515 PMC5898100

[2] Brabec J, Szczepankiewicz F, Lennartsson F, Englund E, Pebdani H, Bengzon J, Knutsson L, Westin CF, Sundgren PC, Nilsson M (2022) Histogram analysis of tensor-valued diffusion MRI in meningiomas: relation to consistency, histological grade and type. NeuroImage Clinical 33:102912. 10.1016/j.nicl.2021.102912 34922122 10.1016/j.nicl.2021.102912 PMC8688887

[3] Chen TC, Zee CS, Miller CA, Weiss MH, Tang G, Chin L, Levy ML, Apuzzo ML (1992) Magnetic resonance imaging and pathological correlates of meningiomas. Neurosurgery 31:1015–1021. 10.1227/00006123-199212000-00005. (**discussion 1021–1012**) 1281915 10.1227/00006123-199212000-00005

[4] Go KO, Lee K, Heo W, Lee YS, Park YS, Kim SK, Lee JH, Jung JM (2018) Cystic meningiomas: correlation between radiologic and histopathologic features. Brain Tumor Research and Treatment 6:13–21. 10.14791/btrt.2018.6.e3 29644810 10.14791/btrt.2018.6.e3 PMC5932295

[5] Hoover JM, Morris JM, Meyer FB (2011) Use of preoperative magnetic resonance imaging T1 and T2 sequences to determine intraoperative meningioma consistency. Surg Neurol Int 2:142. 10.4103/2152-7806.85983 22059137 10.4103/2152-7806.85983 PMC3205511

[6] Huang RY, Bi WL, Griffith B, Kaufmann TJ, la Fougère C, Schmidt NO, Tonn JC, Vogelbaum MA, Wen PY, Aldape K, Nassiri F, Zadeh G, Dunn IF, Meningiomas ICo (2019) Imaging and diagnostic advances for intracranial meningiomas. Neuro-oncol 21:i44–i61. 10.1093/neuonc/noy143.JNeuro-Oncology 30649491 10.1093/neuonc/noy143 PMC6347083

[7] Kashimura H, Inoue T, Ogasawara K, Arai H, Otawara Y, Kanbara Y, Ogawa A (2007) Prediction of meningioma consistency using fractional anisotropy value measured by magnetic resonance imaging. J Neurosurg 107:784. 10.3171/jns-07/10/0784 10.3171/JNS-07/10/0784 17937223

[8] Kim B-W, Kim M-S, Kim S-W, Chang C, Kim O (2011) Peritumoral brain edema in meningiomas : correlation of radiologic and pathologic features. Journal of Korean Neurosurgical Society 49:26–30. 10.3340/jkns.2011.49.1.26 21494359 10.3340/jkns.2011.49.1.26 PMC3070891

[9] Limpastan K, Unsrisong K, Vaniyapong T, Norasetthada T, Watcharasaksilp W, Jetjumnong C (2022) Benefits of combined MRI sequences in meningioma consistency prediction: a prospective study of 287 consecutive patients. Asian journal of neurosurgery 17:614–620. 10.1055/s-0042-1758849 36570751 10.1055/s-0042-1758849 PMC9771632

[10] Miyoshi K, Wada T, Uwano I, Sasaki M, Saura H, Fujiwara S, Takahashi F, Tsushima E, Ogasawara K (2020) Predicting the consistency of intracranial meningiomas using apparent diffusion coefficient maps derived from preoperative diffusion-weighted imaging. J Neurosurg JNS.1. 10.3171/2020.6.Jns20740 10.3171/2020.6.JNS20740 33186907

[11] Ortega-Porcayo LA, Ballesteros-Zebadúa P, Marrufo-Meléndez OR, Ramírez-Andrade JJ, Barges-Coll J, Tecante A, Ramírez-Gilly M, Gómez-Amador JL (2015) Prediction of mechanical properties and subjective consistency of meningiomas using T1–T2 assessment versus fractional anisotropy. World Neurosurg 84:1691–1698. 10.1016/j.wneu.2015.07.018 26188185 10.1016/j.wneu.2015.07.018

[12] Phuttharak W, Boonrod A, Thammaroj J, Kitkhuandee A, Waraasawapati S (2018) Preoperative MRI evaluation of meningioma consistency: a focus on detailed architectures. Clin Neurol Neurosurg 169:178–184. 10.1016/j.clineuro.2018.04.025 29709881 10.1016/j.clineuro.2018.04.025

[13] Romani R, Tang W-j, Mao Y, Wang D-j, Tang H-l, Zhu F-p, Che X-m, Gong Y, Zheng K, Zhong P, Li S-q, Bao W-m, Benner C, Wu J-s, Zhou L-f (2014) Diffusion tensor magnetic resonance imaging for predicting the consistency of intracranial meningiomas. Acta Neurochir 156:1837–1845. 10.1007/s00701-014-2149-y 25002281 10.1007/s00701-014-2149-y

[14] Shiroishi MS, Cen SY, Tamrazi B, D’Amore F, Lerner A, King KS, Kim PE, Law M, Hwang DH, Boyko OB, Liu CS (2016) Predicting meningioma consistency on preoperative neuroimaging studies. Neurosurg Clin N Am 27:145–154. 10.1016/j.nec.2015.11.007 27012379 10.1016/j.nec.2015.11.007 PMC4936899

[15] Simis A, Pires de Aguiar PH, Leite CC, Santana PA Jr, Rosemberg S, Teixeira MJ (2008) Peritumoral brain edema in benign meningiomas: correlation with clinical, radiologic, and surgical factors and possible role on recurrence. Surgical neurology 70:471–477. 10.1016/j.surneu.2008.03.006. (**discussion 477**) 18586307 10.1016/j.surneu.2008.03.006

[16] Sitthinamsuwan B, Khampalikit I, Nunta-aree S, Srirabheebhat P, Witthiwej T, Nitising A (2012) Predictors of meningioma consistency: a study in 243 consecutive cases. Acta Neurochir (Wien) 154:1383–1389. 10.1007/s00701-012-1427-9 22743797 10.1007/s00701-012-1427-9

[17] Smith KA, Leever JD, Hylton PD, Camarata PJ, Chamoun RB (2017) Meningioma consistency prediction utilizing tumor to cerebellar peduncle intensity on T2-weighted magnetic resonance imaging sequences: TCTI ratio. J Neurosurg 126:242–248. 10.3171/2016.1.Jns152329 27058200 10.3171/2016.1.JNS152329

[18] Strand PS, Sagberg LM, Gulati S, Solheim O (2022) Brain infarction following meningioma surgery-incidence, risk factors, and impact on function, seizure risk, and patient-reported quality of life. Neurosurg Rev 45:3237–3244. 10.1007/s10143-022-01840-1 35902426 10.1007/s10143-022-01840-1 PMC9492562

[19] Szczepankiewicz F, Lasič S, van Westen D, Sundgren PC, Englund E, Westin C-F, Ståhlberg F, Lätt J, Topgaard D, Nilsson M (2015) Quantification of microscopic diffusion anisotropy disentangles effects of orientation dispersion from microstructure: applications in healthy volunteers and in brain tumors. Neuroimage 104:241–252. 10.1016/j.neuroimage.2014.09.057 25284306 10.1016/j.neuroimage.2014.09.057 PMC4252798

[20] Takayuki H (2019) Surgical indication and treatment strategy for meningioma. Japanese Journal of Neurosurgery 28:462–469. 10.7887/jcns.28.462

[21] Toh CH, Castillo M (2021) Peritumoral brain edema volume in meningioma correlates with tumor fractional anisotropy but not apparent diffusion coefficient or cerebral blood volume. Neuroradiology 63:1263–1270. 10.1007/s00234-021-02646-6 33533947 10.1007/s00234-021-02646-6

[22] Tropine A, Dellani PD, Glaser M, Bohl J, Plöner T, Vucurevic G, Perneczky A, Stoeter P (2007) Differentiation of fibroblastic meningiomas from other benign subtypes using diffusion tensor imaging. Journal of magnetic resonance imaging : JMRI 25:703–708. 10.1002/jmri.20887 17345634 10.1002/jmri.20887

[23] Watanabe K, Kakeda S, Yamamoto J, Ide S, Ohnari N, Nishizawa S, Korogi Y (2016) Prediction of hard meningiomas: quantitative evaluation based on the magnetic resonance signal intensity. Acta Radiol (Stockholm, Sweden : 1987) 57:333–340. 10.1177/0284185115578323 10.1177/0284185115578323 25824207

[24] Yamaguchi N, Kawase T, Sagoh M, Ohira T, Shiga H, Toya S (1997) Prediction of consistency of meningiomas with preoperative magnetic resonance imaging. Surg Neurol 48:579–583. 10.1016/s0090-3019(96)00439-9 9400639 10.1016/s0090-3019(96)00439-9

[25] Yao A, Pain M, Balchandani P, Shrivastava RK (2018) Can MRI predict meningioma consistency?: a correlation with tumor pathology and systematic review. Neurosurg Rev 41:745–753. 10.1007/s10143-016-0801-0 27873040 10.1007/s10143-016-0801-0 PMC5438899

[26] Yogi A, Koga T, Azama K, Higa D, Ogawa K, Watanabe T, Ishiuchi S, Murayama S (2014) Usefulness of the apparent diffusion coefficient (ADC) for predicting the consistency of intracranial meningiomas. Clin Imaging 38:802–807. 10.1016/j.clinimag.2014.06.016 25082174 10.1016/j.clinimag.2014.06.016

[27] Zada G, Yashar P, Robison A, Winer J, Khalessi A, Mack WJ, Giannotta SL (2013) A proposed grading system for standardizing tumor consistency of intracranial meningiomas. Neurosurgical Focus FOC 35:E1. 10.3171/2013.8.Focus13274 10.3171/2013.8.FOCUS13274 24289117

