# Supplementary material for: Predicting intraoperative meningioma consistency using features from standard MRI sequences: a preoperative evaluation
Source: Acta Neurochir (Wien). 2025 Jun 21;167(1):173. doi: 10.1007/s00701-025-06582-9 (PMC12182493; doi:10.1007/s00701-025-06582-9)

Supplementary material

Predicting Intraoperative Meningioma Consistency Using Features from Standard MRI Sequences: A Preoperative Evaluation

Department of Radiology, Division of Radiology and Nuclear Medicine, Oslo University Hospital, Oslo, Norway (1)

Department of Neurosurgery, Oslo University Hospital, Oslo, Norway (2)

Department of Physics and Computational Radiology, Division of Radiology and Nuclear Medicine, Oslo University Hospital, Oslo, Norway (3)

Institute of Clinical Medicine, Faculty of Medicine, University of Oslo, Oslo, Norway (4)

Donata Biernat, dobier@ous-hf.no, ORCID iD https://orcid.org/0009-0003-5565-831X

Robin Antony Birkeland Bugge, robbir@ous-hf.no, ORCID iD https://orcid.org/0000-0002-6184-6021

Jon Ramm-Pettersen, jramm@ous-hf.no, ORCID iD https://orcid.org/0000-0002-9224-5624

Till Schellhorn, UXSCTI@ous-hf.no, ORCID iD https://orcid.org/0000-0002-5336-0515

Pål Rønning, paroen@ous-hf.no, ORCID iD https://orcid.org/0000-0002-2279-7415

Eirik Helseth, EHELSETH@ous-hf.no, ORCID iD https://orcid.org/0000-0001-5758-6485

Kyrre Eeg Emblem, kemblem@ous-hf.no, ORCID iD https://orcid.org/0000-0002-6580-9519

Karoline Skogen, kaskog@ous-hf.no, ORCID id https://orcid.org/0000-0003-2452-4178

Figure 4: Receiver operating characteristic (ROC) curve showing the diagnostic performance of normalized T2 tumor values from mean tumor ROI in predicting tumor consistency. The area under the curve (AUC) was 0.69, indicating moderate diagnostic accuracy at threshold 1.72, with sensitivity 69%, specificity 69%. Higher values being indicative of soft tumors.

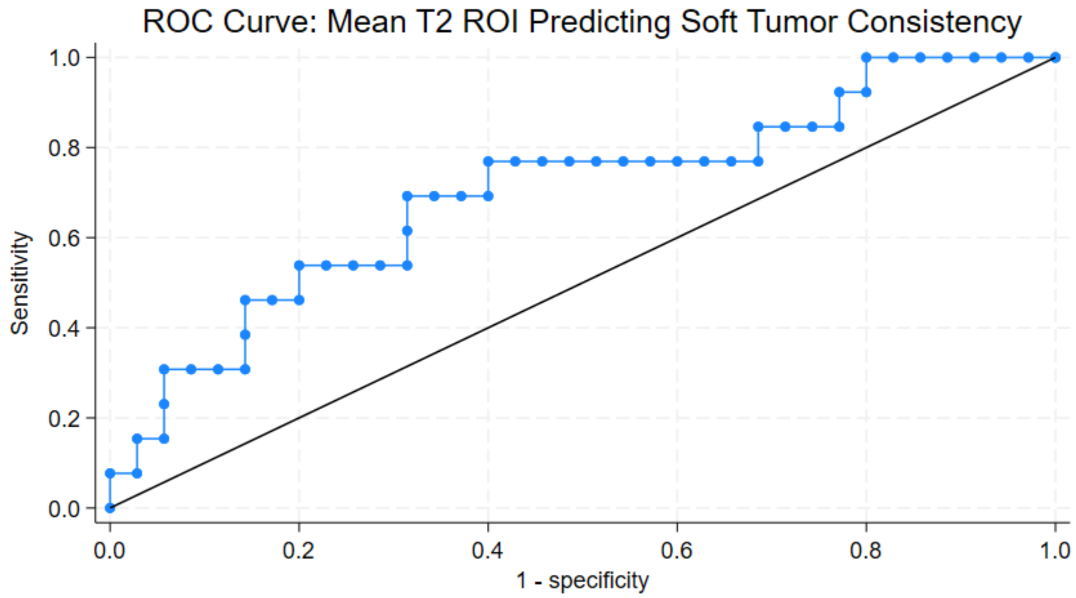

Figure 5: Receiver operating characteristic (ROC) curve showing the diagnostic performance of Tumor/cCP ratio on normalized T2 ROI in predicting tumor consistency. The area under the curve (AUC) was 0.71 indicating moderate diagnostic accuracy at threshold 1.74, with sensitivity 62%, specificity 80%. Higher values being indicative of soft tumors.

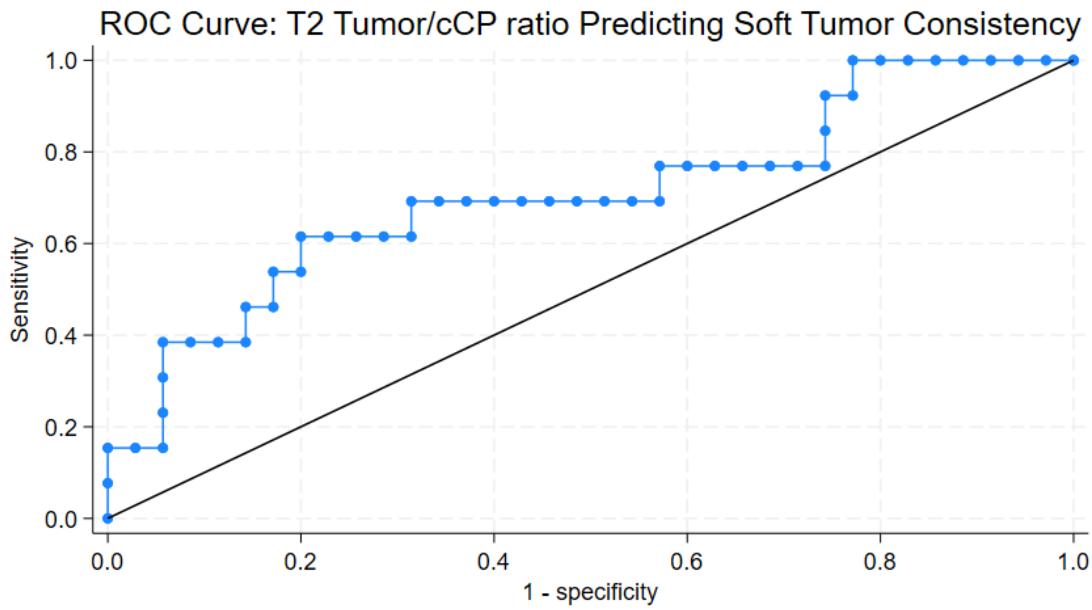

Supplement: Supplementary file 1 — (PDF 171 KB) [file 701_2025_6582_MOESM1_ESM.pdf]
